# Adolescents’ Acceptance of Long-Acting Reversible Contraception After an Educational Intervention in the Emergency Department: A Randomized Controlled Trial

**DOI:** 10.5811/westjem.2020.2.45433

**Published:** 2020-04-21

**Authors:** Tatyana Vayngortin, Lela Bachrach, Sima Patel, Kathleen Tebb

**Affiliations:** * Rady Children’s Hospital, University of California San Diego, Division of Emergency Medicine, San Diego, California; † University of California San Francisco Benioff Children’s Hospital Oakland, Department of Adolescent Medicine, Oakland, California; ‡ University of California San Francisco Benioff Children’s Hospital Oakland, Department of Emergency Medicine, Oakland, California; § University of California San Francisco, Department of Adolescent Medicine, San Francisco, California

## Abstract

**Introduction:**

Adolescents who seek care in the emergency department (ED) are a cohort at increased risk of unintended pregnancy. Although adolescents are interested in learning about pregnancy prevention in the ED, there is a lack of effective educational interventions in this setting. Long-acting reversible contraceptives (LARC) are highly effective and safe in teens, yet are underutilized. This study assessed contraception use among adolescents in the ED and evaluated the impact of an educational video on their interest in and uptake of LARCs.

**Methods:**

We conducted a two-arm randomized controlled trial on a convenience sample of sexually active females 14 to 21 years old in an urban pediatric ED. Participants were randomized to an educational video or standard care. All participants completed a survey and were given an informational card about affiliated teen clinics with the option to schedule an appointment. We assessed pre-post mean differences between control and intervention participants and pre-post differences among intervention participants. Participants were followed three months after their ED visit to examine use of contraception.

**Results:**

A total of 79 females were enrolled (42 control and 37 intervention). The mean age was 17 years, and most were youth of color. The proportion of participants with a prior pregnancy was 18%. Almost all participants reported wanting to avoid pregnancy, yet 18% reported not using contraception at last intercourse. At baseline, 17.7% of participants were somewhat or very interested in the intrauterine device (IUD) or implant. After watching the video, 42.3% were somewhat or very interested in the IUD and 35.7% in the implant. Among those who watched the video, there were significant increases in interest in using an IUD or implant (p<.001). Compared to controls, adolescents who watched the video were also significantly more likely to report wanting an IUD (p<0.001) or implant (p=0.002). A total of 46% were reached for follow-up. Of these, 16% had initiated a LARC method after their ED visit (p=NS).

**Conclusion:**

Most adolescent females in the ED want to avoid pregnancy, but are using ineffective methods of contraception. A brief educational video on LARCs was acceptable to adolescents and feasible to implement in a busy urban ED setting. Adolescents who watched the video had significantly greater interest in using LARCs, but no demonstrated change in actual adoption of contraception.

## INTRODUCTION

While teen pregnancy rates have been declining, the United States continues to have a higher teen pregnancy rate than all other developed nations.^1^ Most (88%) teen pregnancies are unintended^2^; about half of these are due to contraceptive failure resulting from inconsistent or incorrect use, while the rest are due to contraception non-use.^3^ The 2006–2010 National Survey of Family Growth found that less than one-third of females aged 15–19 years consistently used contraception at their last intercourse.^3^,^4^ Condoms continue to be the most common contraceptive method used by adolescents.^4^ While condoms are effective at preventing sexually transmitted infections, they have a high failure rate in preventing pregnancy (18%) based on typical use.^5^ In addition, adolescents were twice as likely to have an unintended pregnancy when using short-acting methods such as oral contraceptive pills (OCP), patch, or ring, compared to adults.^6^

Long-acting reversible contraceptive methods (LARC), such as intrauterine devices (IUD) and contraceptive implants, are highly effective with <1% failure rate and have an excellent safety profile in all age groups, including adolescents.^6^,^7^ They do not require daily adherence or follow-up appointments, and are easy to keep confidential. Therefore, multiple medical organizations recommend including LARCs in contraceptive counseling for adolescents and enhancing access to these methods.^7^–^10^ Despite evidence of the safety and efficacy of LARCs for adolescents, use of these methods remains low among this population (about 3–5% nationally).^11^,^12^

Barriers to adolescents’ use of LARCs include patient-related barriers such as access, cost, and misconceptions, as well as provider-related barriers such as knowledge, attitudes, and clinical competencies.^7^,^8^ There are also disparities in adolescents’ knowledge and access to LARCs based on race/ethnicity, income, and geographic location.^13^ A growing body of evidence indicates that when adolescents have education and access to all of the contraceptive methods, many select LARCs.^14^–^16^ Therefore, increasing education and access to LARCs may increase adolescents’ interest in and use of these methods.

The emergency department (ED) is a potentially valuable setting to provide adolescents with education on pregnancy prevention. Adolescents are less likely to have a primary care provider compared to younger children, and thus may miss opportunities to receive anticipatory guidance on important topics such as reproductive health.^17^ Many adolescents rely on the ED, and those who use it as their primary source of care tend to engage in riskier behaviors, including sex with multiple partners, unprotected sex, and substance use.^17^,^18^ For example, Miller et al surveyed adolescents ages 14–19 in the ED, and found that 45% were sexually active, and of those, 63% reported high-risk behaviors and only one-fourth reported having received contraception counseling.^18^ Multiple studies have found that adolescents are interested in learning about pregnancy prevention during ED visits.^15^, ^19^–^23^ Hoehn et al evaluated in-person, contraceptive counseling in the ED, and found an increase in interest in initiating contraception, especially LARC.^15^ No prior studies have evaluated the use of an educational on contraception in the ED.

**Population Health Research Capsule**

What do we already know about this issue?

Adolescents seeking care in the emergency department (ED) are at increased risk of unintended pregnancy, and are interested in learning about pregnancy prevention in the ED.

What was the research question?

Among sexually active adolescents in the ED, will watching an educational video increase their interest in and use of long-acting reversible contraceptives (LARC)?

What was the major finding of the study?

The study found that an educational video on LARCs was acceptable to adolescents and increased their interest in using LARCs. With 46% followup, we did not demonstrate a change in actual adoption of contraception.

How does this improve population health?

ED-based pregnancy prevention education may increase adolescents’ use of contraception, thereby decreasing unintended pregnancy in this high-risk population.

The objectives of this study were to describe current contraception use among adolescents in the ED, and to evaluate whether showing them a brief educational video about LARCs in the ED would increase their awareness and uptake of LARCs. By improving education and access to contraception when adolescents are already in a healthcare setting, we aim to remove knowledge and access barriers so that high-risk patients can make informed decisions about which contraceptive method is best for them.

## METHODS

### Study Design

We conducted a two-arm, prospective, randomized controlled trial to evaluate the impact of an educational video intervention on adolescents’ attitudes toward and uptake of LARC. The study was approved by the institutional review board of the University of California San Francisco (UCSF) Children’s Hospital Oakland Research Institute, with waived parental and written informed consent.

### Study Setting and Population

The study was conducted at an urban pediatric ED at a freestanding children’s hospital from June 2016–December 2017. This ED has approximately 45,000 annual visits, about 9,000 (20%) of which are adolescents. Among all ED patients, 95% are insured, with 76% of these with government-issued insurance.

### Subject Enrollment

Female patients 14 to 21 years old reporting prior sexual activity were eligible to participate. We excluded patients if they were critically ill, seeking care for a psychiatric chief complaint or sexual assault, not proficient in English, and/or currently using a LARC method. Eligible participants were identified from the ED electronic tracking board. Trained research assistants (RA) approached patients in their ED patient rooms and asked their parent/guardian to step out of the room during study participation. This is standard of care for patients over the age of about 12 years to have the opportunity to speak to a provider without a parent/guardian present.^24^ The RAs explained the study and obtained verbal assent from participants. Participants who agreed to participate were randomized using an online randomization tool (https://www.randomizer.org) to either the control (standard care) or intervention group (video intervention).

### Measures

All participants (control and intervention) completed a baseline paper survey that included questions on demographics, sexual activity, contraception use, pregnancy intention, and interest in using a LARC method (Survey 1). A multidisciplinary team of authors developed the survey tool based on the objectives of the study and previously published, adolescent-survey studies. We pilot tested the survey with 10 adolescent patients, and no significant issues were identified. Interest in LARCs was assessed with a five-point Likert scale (ranging from 1 = not at all interested to 5 = very interested). The survey was self-administered and took approximately five minutes to complete.

The intervention group then watched an eight-minute educational video on LARCs, which was shown on a computer on wheels in the patient’s exam room. This video is publicly available and was created by the UCSF Bixby Center for Global Reproductive Health (https://vimeo.com/123257511). It features adolescents discussing their family planning goals, experiences with LARC methods, and provides information about how each LARC method works, efficacy, and cost. Participants who watched the video also filled out a post-video survey to gather feedback on the video and to assess any change in interest in using LARC, again with a five-point Likert scale (Survey 2). They were asked to rate the video on a five-point Likert scale. Participants were also asked whether they were would be interested in same-day initiation of LARC if it were to be available.

At the end of the survey, participants were asked whether they could be contacted for follow-up and their preferred contact method. All participants were given an informational card about our hospital-affiliated adolescent clinics with an option to have an appointment scheduled by the ED provider. Participants were compensated with a $5 gift card for study completion. If recruitment occurred during business hours, the principal investigator (PI) or RA called the adolescent clinic to schedule the appointment. If recruitment occurred after hours, the PI or RA sent the participant’s contact information to the adolescent clinic scheduler to schedule follow-up the following day.

The PI reviewed the medical records of participants three months after their ED visit to assess whether they had initiated contraception. Participants were also contacted by the PI or RAs via phone, text, or e-mail as per their preference. In the follow-up interview, they were asked about current pregnancy intentions and contraceptive use with a scripted interview tool created by the authors. If they hoped to avoid pregnancy, they were asked if they had initiated a new contraceptive method since their ED visit. If they stated they were not using any contraception, they were asked about barriers to contraception use. The chart abstractors were not blinded to the group allocation.

### Data Analysis

We summarized demographic characteristics of the study population using descriptive statistics. To assess the equivalence of intervention and control participants at baseline, we used t-tests to assess mean differences in age, and chi-square tests to assess differences for race/ethnicity, desire to avoid pregnancy, prior pregnancy, and method used at last intercourse. We analyzed differences in baseline LARC interest (IUD and implant) using the Mann-Whitney U test (nonparametric equivalent of independent sample t-test). To assess the effectiveness of the brief video intervention on LARC interest, Wilcoxon test for paired samples (the nonparametric equivalent of the paired sample t-test) was used to compare pre- and post-video differences in LARC interest. To compare the mean change in LARC interest between intervention and controls, we used the Mann-Whitney U test. We conducted power analyses assuming equal group allocation of 50 participants per group; the difference between means would have to achieve 0.6 standard deviations (SD) to be statistically significant. Data was analyzed using SPSS v25 (IBM SPSS Statistics, Chicago, IL).

## RESULTS

**Figure 1:**
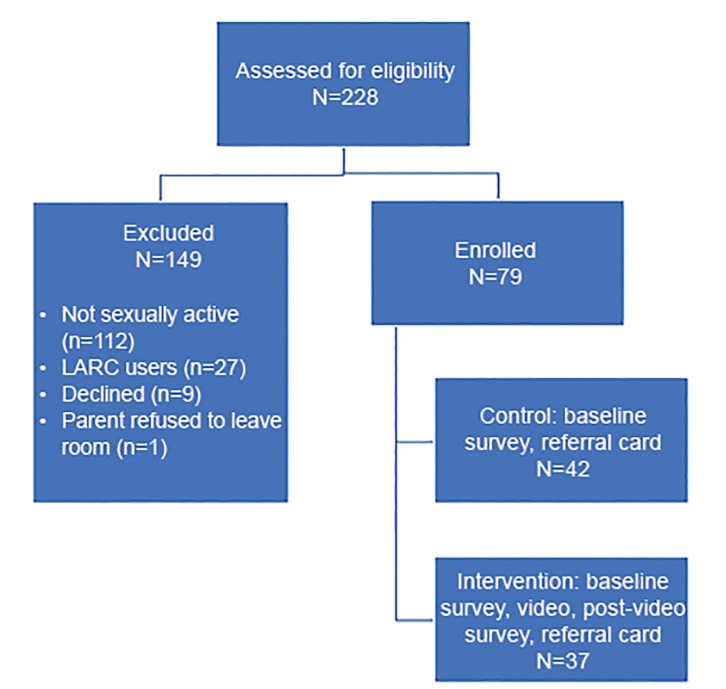
Consolidated Standards of Reporting Trials (CONSORT) diagram demonstrating selection and randomization of subjects in study of adolescent girls’ response to education on pregnancy prevention options in the emergency department. *LARC*, long-acting reversible contraceptive.

Out of 228 potential subjects, 79 were enrolled. We excluded participants for the following reasons: 49% were not sexually active; 25% of sexually active girls were already using a LARC method; 4% declined to participate; and one parent declined to leave the room, so her child was excluded. Of the 79 participants, 42 were randomized to the intervention group, and 37 to the control group, as demonstrated in the Consolidated Standards of Reporting Trials (CONSORT) diagram (Figure 1). This is reported as per CONSORT guidelines.^25^ There were no significant differences in demographics or key variables that could impact outcomes between the control and intervention groups (see Tables 1 and 2). The majority of patients (94%) did not have a gynecologic chief complaint and were seeking care for non-reproductive related problems. Subject characteristics and contraceptive usage by method are described in Table 1. As noted in Table 1, the most frequently used method was a condom and 18% of patients did not use any method at last intercourse, 19% used withdrawal, and 6% used emergency contraception. A total of 18% of patients had a prior pregnancy. A total of 90.14% of participants reported that it was either very or somewhat important to avoid becoming pregnant, and there were no significant differences between intervention and controls (*X*^2^ = 2.25, p = .325). When patients were asked their preferred method with the question “if you could get any method today, which would it be?” answers were variable, as shown in Table 2. There were no significant differences between the control and intervention group in desired contraception method at baseline. The most frequently desired method was condoms (33%), and 15% of patients did not want any method.

At baseline, there was no significant difference between the control and intervention groups in LARC interest. Of the control participants, 29.7% said they were somewhat or very interested in the IUD compared to 21.4% of intervention participants (p = .42). For the implant the baseline difference approached but did not achieve significance with 16.2% of controls expressing interest in an implant compared to 19.0% in the intervention group (p = .07). After watching the video, 42.3% were somewhat or very interested in the IUD and 35.7% in the implant. The pre-post increase was significant for both IUD and implant (p=.001). Compared to controls, the increase in LARC interest was significantly greater for those who watched the video (p<0.001 for IUD, p = 0.002 for implant) (see Table 3). When asked about interest in same-day initiation of LARC if it were available, participants had low interest among all groups. Of the controls, 9.5% and 14.3% would want same-day IUD and implant, respectively. Of the intervention subjects, 14.3% and 13.5% would want same-day IUD and implant respectively, at baseline. After the video, 18.9% and 13.5% would want same-day IUD and implant, respectively. Reasons for not wanting LARC included “not ready,” “not sure,” concerns about pain, and satisfaction with current contraceptive method. We also calculated the mean rating for the following statements: “I learned a lot from the video” – 4.2; “I liked watching the video in the emergency department” – 3.9, and “I would prefer to watch a video like this in another setting other than the emergency department” – 2.9. Subjects also had the opportunity to write subjective comments about the video. Sample comments include the following: “very informative”; “I would recommend other teens watch this”; and “you should do more videos like this so people can learn.”

About half (45.6%) of patients were reached for follow-up, either by direct contact or electronic chart review. Electronic chart review could only capture patients followed in our hospital system, which included 19 patients with documentation on contraception use. Fourteen patients were reached by phone call or text message, and three were reached by e-mail. On phone follow-up, when asked about barriers to using contraception, multiple patients had concerns about side effects such as “it will make me fat.” We had offered to schedule adolescent clinic appointments at the time of the ED visit, but only six participants accepted appointments and only one actually attended. Six patients initiated LARC after their ED visit: two in the control group, and four in the intervention group. Two patients got an IUD, and four got an implant. Of patients who were followed up, 21.6% were using the same hormonal method as during their ED visit (OCP or medroxyprogesterone acetate), 30% were using condoms only, and 30% were not using any method.

## DISCUSSION

This is the first study, to our knowledge, that demonstrated that a brief, video-based educational intervention on contraception shown to patients in a pediatric ED can increase interest in using LARC. Our study found that about half of adolescent females presenting to the ED are sexually active, and a large proportion of them are at risk of unintended pregnancy and in need of contraception. We found a higher rate of LARC use in our population than that reported in the general adolescent population (25% vs 5%, respectively), which is likely due to our adolescent clinic’s efforts to enhance access to LARCs around the same time this study was initiated. Similar to prior literature, our study found that adolescents are interested in ED-based pregnancy prevention education. One advantage of a video-based intervention is that it may be more feasible to implement in a busy ED if a provider does not have sufficient time to engage in comprehensive contraceptive counseling. The study took 10–15 minutes to complete, and most eligible teens agreed to participate. Study participants were highly satisfied with the video and enjoyed watching it.

Our study focused on education on LARC because while these methods are the most effective, they are the least frequently used by adolescents. It is important that adolescents are aware of all contraceptive methods so that they can make an informed decision regarding which method will work best for them. When we asked adolescents their preferred contraceptive method, we found a variety of responses, further demonstrating that patients have various needs and preferences in selecting their contraceptive method. Thus, patient-centered counseling is paramount. Despite significant increases in the desire to use LARC among our study participants, attitudes transitioned from negative to neutral and there was not a significant increase in the actual uptake of LARC. Of subjects who completed follow-up, 16% initiated LARC after their ED visit. However, the majority of patients at follow-up were still using either no method or less effective methods. On follow-up, several patients reported misconceptions about contraceptive side effects.

Chernick et al interviewed adolescent females in the ED about barriers to contraception use, and participants reported concerns of effects on menstruation, weight, fertility, and overall mistrust of contraceptives.^20^ Therefore, providers should strive to address patients’ concerns and misconceptions during contraceptive counseling. ED-based studies involving adolescents frequently demonstrate low follow-up rates, as was seen in our study as well. Chernick et al evaluated referrals with wallet cards for adolescents in the ED to family planning clinics and found no significant difference in follow-up compared to standard discharge instructions.^26^ Hoehn et al offered adolescent females in the ED a scheduled follow-up appointment and found that about half of participants scheduled an appointment, and 40% of those actually attended.^15^

Poor follow-up among adolescents raises the question whether contraception should be initiated in the ED, as this may be their primary healthcare setting. In our study survey, we assessed potential interest in same-day LARC initiation, and one-third of our participants were interested. Miller et al found that two-thirds of adolescents surveyed in the ED were interested in same-day contraception initiation, including one-third interested in LARC.^21^ Hoehn et al also asked adolescents who had missed their appointments whether they would have started contraception during their ED visit if it had been offered, and 77% said yes.^15^ Offering same-day contraception in the ED may decrease unintended pregnancy, but further research is needed to assess the feasibility and acceptance of this. Moreover, offering contraception counseling in the ED is provider-dependent, and may face provider-level barriers including time, training, and motivation.

## LIMITATIONS

The study has several limitations. While our sample size was small, it was sufficient to identify significant differences between our control and intervention groups in interest in using LARC. However, the study was not powered to detect differences in actual uptake of LARC. The study was conducted at a single site, an urban children’s hospital, so our results may not be generalizable to all settings. RAs were only available for limited time periods (ie, a summer research internship), and recruited patients during afternoon-early evening hours on weekdays. The PI also recruited patients at various times when available. The PI and RAs were not blinded to group allocation when conducting follow-up calls and chart review. The survey and video were only available in English, which excluded non-English speaking patients.

Additionally, survey studies are subject to social desirability bias. About half of our patients were lost to follow-up, so our results may have been different if we had follow-up data on all initial participants. Since most adolescent ED visits occurred in the evenings after school, patients had to be contacted the following day to schedule the appointment. This may have been one reason contributing to less follow-up if patients could not be reached. Also, some patients may have followed up with their primary care provider or at another clinic rather than our adolescent clinic. There were concomitant LARC initiatives in our hospital around the time of this study, for example availability of LARC in our adolescent clinic.

## CONCLUSION

Most adolescent females in the pediatric ED want to avoid pregnancy, yet many are using ineffective or no contraception. They are interested in a wide range of family planning methods; therefore, it is important to provide them with comprehensive, patient-centered, contraceptive counseling. We found that a brief educational video on LARCs was acceptable to adolescents and successfully implemented in a busy ED setting. Adolescents who watched the video were significantly more interested in using LARCs; however, LARC initiation remained low. Future studies are needed to determine the most effective method of providing contraception education to adolescents to improve contraception uptake and access.

## Supplementary Information





## Figures and Tables

**Table 1:** Demographics of adolescent girls who responded to survey regarding baseline contraception.

Variables	Control (N=42)	Intervention (N=37)	P-value
Mean age	17.3	16.8	p = .853
Ethnicity	n (%)	n (%)	p = .805
African-American	20 (47.6)	17 (45.9)	
Hispanic	14 (33.3)	14 (37.8)	
Multi-ethnic	5 (11.9)	2 (5.4)	
Caucasian	3 (7.1)	3 (8.1)	
Asian/Pacific-Islander	0 (0)	1 (2.7)	
Have a primary care doctor	31 (73.8)	33 (89.2)	p = .084
Prior pregnancy*	7 (16.7)	7 (18.9)	p = .789
>1 pregnancy	1 (14.3)	2 (28.6)	
Therapeutic abortion	4 (57.1)	6 (85.7)	
Spontaneous abortion	2 (28.6)	3 (42.9)	
Birth	1 (14.3)	2 (28.6)	
Method used at last intercourse:			
Condoms	22 (52.4)	20 (54.1)	p = .537
Withdrawal or none	14 (33.3)	7 (18.9)	p = .352
Multiple methods	9 (21.4)	11 (29.7)	p = .540
Medroxyprogesterone acetate	6 (14.3)	7 (18.9)	p = .391
Oral contraceptive pill	5 (11.9)	4 (10.8)	p = .589
Emergency contraception	2 (4.8)	3 (8.1)	p = .434
NuvaRing	3 (7.1)	0 (0)	p = .147
Patch	0	0	n/a

* For patients who had a history of prior pregnancy, the values listed (ie, therapeutic abortion) refer to the proportion of prior pregnancies. Data was dichotomized as Yes/No.

**Table 2:** Desired contraceptive method at baseline.

Contraceptive method	n (%)	n (%)	P-value
Condoms	16 (38.1)	10 (27)	p = .420
OCP	10 (23.8)	8 (21.6)	p = .556
Medroxyprogesterone acetate	8 (19)	7 (18.9)	p = .368
None	6 (14.3)	5 (13.5)	p = .620
Implant	3 (7.1)	5 (13.5)	p = .265
NuvaRing	3 (7.1)	4 (10.8)	p = .580
Patch	5 (11.9)	2 (5.4)	p = .265
IUD	3 (7.1)	2 (5.4)	p = .434
Emergency contraception	1 (2.4)	0 (0)	p = .542

*IUD*, intrauterine device; *OCP*, oral contraceptive pills.

**Table 3:** Mean Difference in Interest in LARC use.

LARC Method	Control Mean Difference (SD)	Intervention Mean Difference (SD)	(95% CI, p-value)
IUD	0 (0.0)	0.686 (1.13)	(0.297, 1.07, p=.001)
Implant	0 (0.0)	0.514 (0.96)	(0.193, 0.83, p=.003)

*CI*, confidence interval; *IUD*, intrauterine device; *LARC*, long-acting reversible contraceptive; *SD*, standard deviation.
