# Supplementary material for: Adolescents’ Acceptance of Long-Acting Reversible Contraception After an Educational Intervention in the Emergency Department: A Randomized Controlled Trial
Source: West J Emerg Med. 2020 Apr 21;21(3):640–6. doi: 10.5811/westjem.2020.2.45433 (PMC7234691; doi:10.5811/westjem.2020.2.45433)
Supplement: Supplementary file 1 [file wjem-21-640-s001.docx]

**Demographics Survey**

1. Age:
2. Race/Ethnicity:
   1. White
   2. African-American
   3. Hispanic/Latino
   4. Asian/Pacific-Islander
   5. Multi-ethnic
   6. Other
3. Why are you in the emergency department today? (please write your answer)
4. Have you seen a doctor in clinic in the last 12 months?
   1. Yes
   2. No
5. Have you ever had sexual intercourse (by sex, we mean penis in vagina)?
   1. Yes
   2. No

**If you answered No, please stop the survey. Thank you for your help**

**If you answered Yes, please continue the survey**

1. When was the last time you had sex?

7. Which methods of birth control have you heard of? (Circle all the methods you have heard of)
a. Condoms
b. Birth control pills
c. Patch
d. Nuva Ring
e. Depo shot
f. Intrauterine device (IUD), such as Mirena, Paraguard, Skyla, copper
g. Arm implant, such as Nexplanon

h. Pulling out/withdrawal

i. Emergency contraception (Plan B, Ella)

j. Calendar/rhythm method

8. Which methods of birth control have you tried? (Circle all that are true)

a. Condoms
b. Birth control pills
c. Patch
d. Nuva Ring
e. Depo shot
f. Intrauterine device (IUD), such as Mirena, Paraguard, Skyla, copper
g. Arm implant, such as Nexplanon

h. Pulling out/withdrawal

i. Emergency contraception (Plan B, Ella)

j. Calendar/rhythm method

9. Which method did you use the last time you had sex? (you may select more than one)
a. I did not use any method
b. Condoms
c. Birth control pills
d. Patch
e. Nuva Ring
f. Depo shot
g. Intrauterine device (IUD), such as Mirena, Paraguard, Skyla, copper

h. Arm implant, such as Nexplanon

i. Pulling out/withdrawal

j. Emergency contraception (Plan B, Ella)

k. Calendar/rhythm method

10. Have you ever been pregnant?

- 1. Yes🡪If you answered yes, please list the number of pregnancies you have had:

number of abortions:

number of births:

number of miscarriages:

- 1. No

11.Thinking about your life right now, how important is it to you to avoid getting pregnant?

- 1. Very important to avoid getting pregnant
  2. Somewhat important to avoid getting pregnant
  3. “If it happens, it happens”
  4. Hoping to get pregnant
  5. Very much hoping to get pregnant

12. If you could get any method of birth control today, which one would you want?

a. Condoms
b. Birth control pills
c. Patch
d. Nuva Ring
e. Depo shot
f. IUD or intrauterine device (Mirena, Paraguard, Skyla)
g. Implant (Nexplanon) (in your arm)

h. Emergency Contraception (Plan B, Ella)

i. None of these

13. Do you have any friends that use an IUD?

a. Yes

b. No

14. How interested would you be in getting an IUD?

a. Very interested

b. Somewhat interested

c. Neutral

d. Not very interested

e. Not at all interested

f. I already have an IUD

15. Do you have any friends that use an arm implant?

a. Yes

b. No

16. How interested would you be in getting an arm implant?

a. Very interested

b. Somewhat interested

c. Neutral

d. Not very interested

e. Not at all interested

f. I already have an implant

17. If it was possible to get an IUD in the emergency department today, would you want it?

a. Yes

b. No- if no, please write why not?

18. If it was possible to get an Implant in the emergency department today, would you want it?

a. Yes

b. No- if no, please write why not?

19. Would you like us to help you make an appointment in our Adolescent clinic to discuss your family planning needs further?

a. Yes🡪**If you answer yes, please let us know when we collect this survey**

b. No

20. Can we reach you in 3 months to ask a few more questions?

a. Yes

b. No

Please write 2 ways to reach you (for example, call, text, e-mail)
