# Supplementary material for: Adolescents’ Acceptance of Long-Acting Reversible Contraception After an Educational Intervention in the Emergency Department: A Randomized Controlled Trial
Source: West J Emerg Med. 2020 Apr 21;21(3):640–6. doi: 10.5811/westjem.2020.2.45433 (PMC7234691; doi:10.5811/westjem.2020.2.45433)
Supplement: Supplementary file 2 [file wjem-21-640-s002.doc]

**Post-Video Survey**

Please rate how much you agree or disagree with the following statements (1: strongly disagree, 2: disagree, 3: neutral, 4: agree, 5: strongly agree)

1. I learned a lot from the video

1 2 3 4 5

1. I liked watching the video in the emergency department

1 2 3 4 5

1. I would prefer to watch a video like this in another setting other than the emergency department

1 2 3 4 5

1. After watching the video, how interested would you be in getting an IUD?
2. Very interested
3. Somewhat interested
4. Don’t know
5. Not very interested
6. Not at all interested
7. After watching the video, how interested would you be in getting an Implant?
8. Very interested
9. Somewhat interested
10. Don’t know
11. Not very interested
12. Not at all interested
13. Please write any comments or questions about the video
14. If it was possible to get an IUD in the emergency department today, would you want it?
15. Yes
16. No- if no, please write why not?
17. If it was possible to get an Implant in the emergency department today, would you want it?
    1. Yes
    2. No- if no, please write why not?
18. Would you like us to help you make an appointment in Adolescent Clinic to discuss your family planning needs further?
19. Yes🡪**if you answered yes, please let us know when we collect the survey**
20. No- if no, please write why not?
